# Quantification of CYP3A and Drug Transporters Activity in Healthy Young, Healthy Elderly and Chronic Kidney Disease Elderly Patients by a Microdose Cocktail Approach

**DOI:** 10.3389/fphar.2021.726669

**Published:** 2021-09-17

**Authors:** Punyabhorn Rattanacheeworn, Stephen J Kerr, Wonngarm Kittanamongkolchai, Natavudh Townamchai, Suwasin Udomkarnjananun, Kearkiat Praditpornsilpa, Thanundorn Thanusuwannasak, Udomsak Udomnilobol, Suree Jianmongkol, Boonsri Ongpipattanakul, Thomayant Prueksaritanont, Yingyos Avihingsanon, Pajaree Chariyavilaskul

**Affiliations:** ^1^ Clinical Pharmacokinetics and Pharmacogenomics Research Unit, Chulalongkorn University, Bangkok, Thailand; ^2^ Biostatistics Excellence Center, Faculty of Medicine, Chulalongkorn University, Bangkok, Thailand; ^3^ Maha Chakri Sirindhorn Clinical Research Center Under the Royal Patronage, Research Affairs, Faculty of Medicine, Chulalongkorn University, Bangkok, Thailand; ^4^ Division of Nephrology, Department of Medicine, Faculty of Medicine, Chulalongkorn University, Bangkok, Thailand; ^5^ Excellent Center of Geriatrics, King Chulalongkorn Memorial Hospital, Thai Red Cross Society, Bangkok, Thailand; ^6^ Chulalongkorn University Drug and Health Products Innovation Promotion Center, Faculty of Pharmaceutical Sciences, Chulalongkorn University, Bangkok, Thailand; ^7^ Chulalongkorn University Drug Discovery and Drug Development Research Center, Chulalongkorn University, Bangkok, Thailand; ^8^ Department of Pharmacology and Physiology, Faculty of Pharmaceutical Sciences, Chulalongkorn University, Bangkok, Thailand; ^9^ Department of Biochemistry and Microbiology, Faculty of Pharmaceutical Sciences, Chulalongkorn University, Bangkok, Thailand; ^10^ Department of Pharmacology, Faculty of Medicine, Chulalongkorn University, Bangkok, Thailand

**Keywords:** pharmacokinetics, microdose cocktail, cytochrome P450, drug transporters, elderly, chronic kidney disease

## Abstract

**Background:** Ageing and chronic kidney disease (CKD) affect pharmacokinetic (PK) parameters. Since mechanisms are related and remain unclear, cytochrome P450 (CYP) 3A and drug transporter activities were investigated in the elderly with or without CKD and compared to healthy adults using a microdose cocktail.

**Methods:** Healthy young participants (*n* = 20), healthy elderly participants (*n* = 16) and elderly patients with CKD (*n* = 17) received, in study period 1, a single dose of microdose cocktail probe containing 30 µg midazolam, 750 µg dabigatran etexilate, 100 µg atorvastatin, 10 µg pitavastatin, and 50 µg rosuvastatin. After a 14-day wash-out period, healthy young participants continued to study period 2 with the microdose cocktail plus rifampicin. PK parameters including area under the plasma concentration-time curve (AUC), maximum plasma drug concentration (C_max_), and half-life were estimated before making pairwise comparisons of geometric mean ratios (GMR) between groups.

**Results:** AUC and C_max_ GMR (95% confidence interval; CI) of midazolam, a CYP3A probe substrate, were increased 2.30 (1.70–3.09) and 2.90 (2.16–3.88) fold in healthy elderly and elderly patients with CKD, respectively, together with a prolonged half-life. AUC and C_max_ GMR (95%CI) of atorvastatin, another CYP3A substrate, was increased 2.14 (1.52–3.02) fold in healthy elderly and 4.15 (2.98–5.79) fold in elderly patients with CKD, indicating decreased CYP3A activity related to ageing. Associated AUC changes in the probe drug whose activity could be modified by intestinal P-glycoprotein (P-gp) activity, dabigatran etexilate, were observed in patients with CKD. However, whether the activity of pitavastatin and rosuvastatin is modified by organic anion transporting polypeptide 1B (OATP1B) and of breast cancer resistance protein (BCRP), respectively, in elderly participants with or without CKD was inconclusive.

**Conclusions:** CYP3A activity is reduced in ageing. Intestinal P-gp function might be affected by CKD, but further confirmation appears warranted.

**Clinical Trial Registration:**http://www.thaiclinicaltrials.org/ (TCTR 20180312002 registered on March 07, 2018)

## Introduction

Chronic kidney disease (CKD) is a significant public health problem worldwide, impacting national health expenditures and patient morbidity and mortality (United Nations, 2020). The overall prevalence of CKD in the Thai population is remarkably high (17.5% of the population), and the prevalence increases with advancing age (Ingsathit et al., 2009). Thailand is currently in a significant demographic shift, with a rapid increase in the ageing population and the elderly, defined in this study as those aged >60 years (World Health Organization, 2015). Due to physiological changes in the elderly, such as a reduction in renal mass, renal blood flow, glomerular filtration rate, and tubular function, the ability of renal drug elimination can be declined (Hutchison and O'Brien, 2007). For renally excreted drugs, ageing causes changes in the pharmacokinetics of drugs leading to unexpected drug efficacy and safety (Tieu et al., 2016). Age-related alterations in liver function may also change drug metabolism and disposition through alteration of the cytochrome P450 (CYP) enzymes and drug transporter activity (Eldesoky, 2007; Nolin et al., 2008; Klotz, 2009; Yeung et al., 2014). Because of multiple comorbid conditions, polypharmacy is also more common in ageing patients. Physiologic changes in drug metabolism and clearance in the elderly increase the risk of adverse drug reactions and drug-drug interactions in this patient group (Dagli and Sharma, 2014; American Geriatrics Society Beers Criteria®Update Expert Panel, 2019).

Cytochrome P450 3A (CYP3A), the most abundant isozyme in the liver and intestine (Blech et al., 2008), plays an important role in metabolizing several drugs and is commonly implicated in pharmacokinetic drug-drug interactions (Lynch and Price, 2007). For the past decade, increasing numbers of clinically important drug-drug interactions mediated by drug transporters, including intestinal P-glycoprotein (intestinal P-gp), organic anion-transporting polypeptide 1B (OATP1B), and breast cancer resistance protein (BCRP), have been reported (Giacomini et al., 2010).

Clinical studies using probe substrate cocktails are recognized by both the United States Food and Drug Administration (United States FDA) and the European Medicines Agency (EMA) to evaluate the activity of multiple CYP enzymes and drug transporters simultaneously (European Medicines Agency, 2012; Center for Drug Evaluation and Research (CDER) U.S. Department of Health and Human Sciences Food and Drug Administration, 2020). A cocktail comprising a sub-therapeutic dose (microdose) of 5 drugs was recently validated in healthy subjects (Prueksaritanont et al., 2017). The cocktail drugs include 1) midazolam (MDZ), a specific and selective substrate for CYP3A; 2) dabigatran etexilate (DABE), a selective substrate for intestinal P-gp; 3) pitavastatin (PTV), a relatively selective substrate for OATP1B; 4) rosuvastatin (RSV), a substrate of BCRP and OATP1B; and 5) atorvastatin (ATV), a substrate of CYP3A, OATP1B, BCRP, and P-gp. This cocktail was subsequently studied in patients with CKD, and the study results suggested that CKD reduces intestinal P-gp and BCRP activity (Tatosian et al., 2020). However, whether the observed changes were due solely to CKD or as a consequence of advancing age, remains inconclusive.

This study aimed to evaluate age- versus CKD-related changes in the activity of CYP3A and the drug transporters, P-gp, OATP, and BCRP, using a microdose cocktail in the elderly with and without CKD compared to young healthy participants.

## Materials and Methods

This study was a clinical pharmacokinetic study (Clinical trial registration number: TCTR 20180312002 registered on March 07, 2018). The study was approved by the Institutional Review Board of the Faculty of Medicine, Chulalongkorn University, Bangkok, Thailand, and was conducted in accordance with the Declaration of Helsinki and Good Clinical Practice Guidelines. Written informed consent was obtained from all participants before the start of the study. The nomenclature of enzymes and drug transporters conformed to the International Union of Basic and Clinical Pharmacology/British Pharmacological Society (IUPHAR/BPS) Guide to Pharmacology Nomenclature Classification (Alexander et al., 2017).

### Participants

Participants were classified into three groups; group 1 (healthy young participants) aged 20–40 years with estimated glomerular filtration rate (eGFR) ≥90 ml/min/1.73 m^2^, group 2 (healthy elderly participants) aged ≥60 years with eGFR >60 ml/min/1.73 m^2^, and group 3 (elderly patients with CKD) aged ≥60 years with eGFR 15–60 ml/min/1.73 m^2^, diagnosed as patients with CKD by attending nephrologists. The pre-specified inclusion/exclusion criteria were fully described in Supplementary Table S1. Modification of Diet in Renal Disease (MDRD) eGFR for Thai was calculated according to Praditpornsilpa et al. (2011).

**FIGURE 1:**
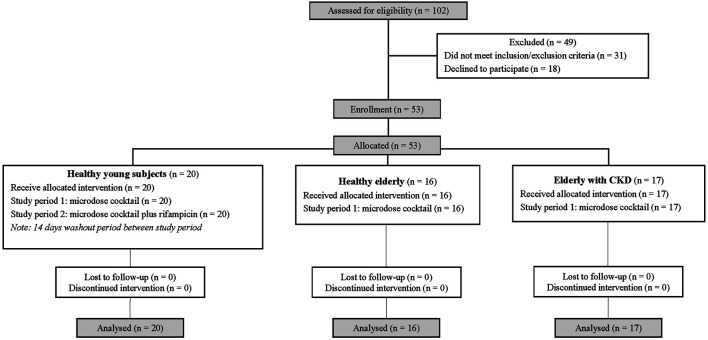
Study flow diagram.

We assumed that the area under the plasma concentration-time curve (AUC) of MDZ between healthy young participants and the elderly participants would differ by 70% with a common standard deviation of 11.84, based on a previous study using the same microdose cocktail (Prueksaritanont et al., 2017). Under these assumptions, eleven participants per study group would give 90% power to detect these differences at a 2-sided significance level of 5%. Therefore, a total of fifty-three participants was enrolled. Twenty, sixteen, and seventeen participants were healthy young adults, healthy elderly, and elderly patients with CKD, respectively (Figure 1).

Healthy young participants were recruited from the volunteers’ database of Maha Chakri Sirindhorn Clinical Research Center under the Royal Patronage, Faculty of Medicine, Chulalongkorn University, Bangkok, Thailand. Healthy elderly participants were recruited from out-patients of the Comprehensive Geriatric Clinic, King Chulalongkorn Memorial Hospital, Thai Red Cross Society, Bangkok, Thailand. Elderly patients with CKD were recruited from an out-patient of the Nephrology Clinic, King Chulalongkorn Memorial Hospital, by nephrologists in the study team.

### Clinical Study and Biological Sample Collection Procedures

All participants were advised to refrain from xanthine-containing beverages, citrus juice, herbal and dietary supplements, and products containing St. John’s wort for 72 h before study start, until study conclusion. In addition, healthy young and healthy elderly participants abstained from any drug intake for at least two weeks before the study date. Elderly patients with CKD continued their medications as usual, except for PTV, ATV, or RSV, which were stopped or switched to the equivalent dose of simvastatin at least two weeks before the study date. The concomitant drugs in elderly patients with CKD were also examined for potential drug-drug interaction with probe substrates in the microdose cocktail.

The powder formulation of microdose cocktail containing 30 µg of MDZ, 750 µg of DABE, 10 µg of PTV, 100 µg of ATV, and 50 µg of RSV, was prepared by a single pharmacist trained in the study procedures at the Pharmacy Department, King Chulalongkorn Memorial Hospital. The microdose cocktail was dissolved with 10 ml of water into a solution before immediate oral administration.

All participants received a single oral dose of the microdose cocktail after 10 h of an overnight fast (study period 1). Venous blood samples were collected into EDTA tubes at predose (0) and 0.33, 0.67, 1, 1.5, 2, 3, 4, 6, 8, 10, 12, 24, 36, and 48 h post-dose. Urine samples were collected at predose (0) and then between 0–4 h, 4–8 h, 8–12 h, 12–24 h, 24–36 h, and 36–48 h post-dose. Plasma and urine samples were aliquoted and kept at −80°C until analysis. Samples for statins analysis were treated immediately with 1 M ammonium buffer (pH 5) at a ratio of 5/100 (buffer/plasma) to prevent interconversion. All participants stayed at the clinical study site for at least 12 h post-dose and returned for subsequent blood sample collections.

After a 2-week wash-out period, the healthy young participants continued to study period 2. On this occasion, participants received another single oral microdose cocktail together with 450 mg of rifampicin (RIF), a well-known OATP1B, BCRP, and intestinal P-gp inhibitor, to evaluate the magnitude of drug-drug interactions between RIF and microdose cocktail in Thai subjects, as compared to the earlier study in Caucasian (Prueksaritanont et al., 2017). Blood and urine samples on study period 2 were collected as previously described in study period 1. The occurrence of any adverse event was carefully monitored throughout the study.

It has to be noted that during the final part of the study period, the MDZ tablet was not available in Thailand. Hence, an intravenous form of MDZ was used in 4 elderly patients with CKD instead. After dehydrating, the solution was prepared to power for the preparation of the microdose cocktail. Despite limited power, pharmacokinetic parameters between powder and solution of MDZ were similar (Supplementary Table S2).

### Bioanalysis

Plasma concentrations of MDZ, dabigatran (DABI) which is an active metabolite of DABE (Blech et al., 2008), PTV, pitavastatin lactone (PTV-lactone), ATV, 2-hydroxy atorvastatin (2-OH-ATV), 4-hydroxy atorvastatin (4-OH-ATV), RSV, RIF, and stable isotope-labeled internal standards were quantified by a validated liquid chromatography-tandem mass spectrometry assay as previously described (Prueksaritanont et al., 2017). In addition, urine concentrations of DABI and RSV were also measured.

MDZ, statins, and RIF were measured using reverse-phase liquid chromatography. Hydrophilic interaction liquid chromatography with tandem mass spectrometric detection employing a turbo ion spray interface in positive ion mode was used for DABI. The lower limit of quantitation (LLOQ) was 1 pg/ml for MDZ, 20 pg/ml for DABI, and 5 pg/ml for statins and their metabolites.

### Genotype Analysis

The Clinical Pharmacogenetics Implementation Consortium (CPIC) guideline recommends simvastatin drug monitoring and dose adjustment in patients based on solute carrier organic anion transporter 1B1 (*SLCO1B1*) genotypic test results, as statins levels are affected by single nucleotide polymorphisms (SNPs) in drug transporters (Ramsey et al., 2014). Therefore, we genotyped participants for *SLCO1B1* and ATP Binding Cassette Subfamily G2 (*ABCG2*) genes to adjust for this possible confounding factor (Keskitalo et al., 2009; Romaine et al., 2010; Ramsey et al., 2014). The genotyping methods together with the genetic variation in *SLCO1B1* c.521T > C (rs4149056), c.388A > G (rs2306283), g.-11187G > A (rs4149015), and *ABCG2* c.421C > A (rs2231142) of all study participants have previously been reported (Rattanacheeworn et al., 2020). In addition, as genetic variation in CYP3A was reported in the Thai population (Mauleekoonphairoj et al., 2020), genotyping for CYP3A5*3 c.6986A > G (rs776746) was also carried out in all participants. Supplementary Table S3 summarized the distribution and the genotyping method of CYP3A5*3 in this study*.*

### Pharmacokinetic Analysis

Pharmacokinetic data were analyzed using noncompartmental methods. Maximum plasma concentration (C_max_) and time to maximum plasma concentration (T_max_) were directly obtained from the individual plasma drug concentration profiles versus the time curve. The area under the plasma concentration-time curve from time zero to the last point with measurable concentration (AUC_0-last_) was calculated using the log-linear trapezoidal rule. AUC_0-inf_ was obtained from the summation of AUC_0-last_ and the last observed quantifiable concentration (C_last_)/elimination rate constant (K_el_). K_el_ was estimated by log-linear least square regression of the terminal part of the plasma concentration versus time curve. Half-life (T_1/2_) was calculated using a natural logarithm of 2 (ln2) divided by K_el_. Oral clearance (CL/F with weight normalization) was also calculated as dose divided by AUC_0-last_. The cumulative amount in urine (A_e_) was measured and divided by AUC_0-last_ to estimate renal clearance (CL_R_) in DABI and RSV. Additionally, the theoretical AUC_0-last_ ratios (AUCR) in AUC_0-last_ of healthy elderly and elderly patients with CKD over AUC_0-last_ of healthy young participants were calculated for DABI and RSV, assuming no change in bioavailability (F). The calculated AUCRs were compared to the observed AUCRs to confirm the effects of ageing and/or CKD on intestinal P-gp and BCRP functions.

### Statistical Analysis

Statistical analysis was performed with STATA version 15.0 (Stata Corp, College Station, TX, United States). Graphs were created by GraphPad Prism version 8.0 (GraphPad Software, Inc., San Diego, CA, United States). Demographic data are presented as median (interquartile range; IQR) or frequency (%) for continuous and categorical data, respectively. A Kruskal-Wallis test analyzed formal comparisons of continuous characteristics between groups, and when differences were found, pairwise comparisons were further investigated with a Wilcoxon rank-sum test. Finally, categorical characteristics were compared with a Chi-square or Fisher’s exact test as appropriate.

The geometric mean (GM) with 95% confidence interval (95%CI) was calculated for AUC_0-last_, AUC_0-inf_, C_max_, CL/F, and CL_R_. Regression techniques with an outcome of the natural logarithm of each parameter were developed for these parameters, and pairwise comparisons were made between groups. The relevant model parameters were then exponentiated to obtain geometric mean ratios (GMR) and corresponding 95% confidence interval (95%CI). T_max_ and T_1/2_ were reported as median (IQR), and pairwise comparisons were made with a Kruskal-Wallis test/Wilcoxon test for demographic parameters. Pre-RIF versus post-RIF comparisons in healthy young participants were assessed using Generalized Estimating Equation (GEE). All *p*-values were adjusted for multiple comparisons using a Bonferroni correction. Multivariable models were used to adjust group differences for potential confounders, including gender, body weight, alanine aminotransferase, aspartate aminotransferase, total bilirubin, direct bilirubin, albumin, and genotype of *SLCO1B1* and *ABCG2* genes.

## Results

### Baseline Characteristics

Baseline characteristics between three groups of participants were comparable except for age, body weight, and renal function (Table 1). The median age was 30, 65, and 74 years and the median eGFR corrected for body surface area (BSA) was 112, 95, and 33 ml/min for healthy young participants, healthy elderly, and elderly patients with CKD, respectively (Table 1). From a list of concomitant drugs in elderly patients with CKD (Supplementary Table S4), no potential pharmacokinetic drug-drug interaction to probe substrates used in this study was noted.

**TABLE 1:** Baseline characteristics.

Parameters	Healthy young adults	Healthy elderly	Healthy young adults *vs.* healthy elderly	Elderly with CKD	Healthy young adults *vs.* elderly with CKD	Healthy elderly *vs.* elderly patients with CKD
N	20	16		17		
Male/female (n/n)	8/12	4/12		13/4		
Age (year)	30 (28–32)	65 (62–67)	*p* < 0.001	74 (67–77)	*p* < 0.001	ns
Body weight (kg)	54.2 (46.8–65.5)	60.4 (52.4–65.0)	ns	66.5 (60.8–70.3)	*p* = 0.0140	ns
**Biochemistry**						
Blood urea nitrogen (mg/dL)	10 (10–12)	11 (10–14)	ns	26 (21–37)	*p* < 0.001	*p* < 0.001
Serum creatinine (g/dL)	0.7 (0.7–0.9)	0.7 (0.6–0.8)	ns	2.2 (1.8–2.9)	*p* < 0.001	*p* < 0.001
eGFR^a^ corrected by BSA (mL/min)	112 (106–118)	95 (86–110)	ns	33 (24–40)	*p* < 0.001	*p* < 0.001
Parathyroid hormone (pg/mL)	55 (45–60)	63 (49–76)	ns	113 (76–168)	*p* < 0.001	*p* < 0.001
Fasting plasma glucose (mg/dL)	85 (81–93)	95 (90–102)	ns	98 (88–105)	ns	ns
Total protein (g/dL)	7.8 (7.5–7.9)	7.7 (7.3–7.8)	ns	7.6 (7.1–7.7)	ns	ns
Albumin (g/dL)	4.5 (4.3–4.6)	4.3 (4.3–4.5)	ns	4.3 (4.2–4.4)	ns	ns
Total bilirubin (mg/dL)	0.6 (0.5–0.8)	0.6 (0.6–0.9)	ns	0.6 (0.5–0.7)	ns	ns
Direct bilirubin (mg/dL)	0.2 (0.2–0.4)	0.3 (0.2–0.3)	ns	0.3 (0.2–0.3)	ns	ns
Aspartate aminotransferase (U/L)	16 (15–20)	22 (19–24)	ns	18 (16–26)	ns	ns
Alanine aminotransferase (U/L)	15 (13–20)	19 (16–22)	ns	19 (16–25)	ns	ns
Alkaline phosphatase (U/L)	54 (47–66)	67 (56–75)	ns	64 (53–73)	ns	ns
Total cholesterol (mg/dL)	203 (176–225)	242 (209–270)	ns	167 (152–177)	ns	ns
Triglyceride (mg/dL)	87 (61–107)	130 (94–191)	ns	91 (87–141)	ns	ns
**Co-morbidity**						
Hypertension	−	−		14 (82)		
Dyslipidemia	−	−		11 (65)		
Diabetes Mellitus	−	−		11 (65)		
Coronary heart disease	−	−		1 (6)		
Chronic kidney disease	−	−		17 (100)		
Osteoarthritis	−	2 (13)		1 (6)		
Benign prostatic hypertrophy	−	1 (6)		1 (6)		
Gout	−	1 (6)		1 (6)		

Data are presented in the median (interquartile range) unless otherwise stated. CKD: chronic kidney disease; eGFR: estimated glomerular filtration rate; BSA: body surface area; ns: non-significant.

a Modification of Diet in Renal Disease estimated glomerular filtration rate for Thai formula (Praditpornsilpa et al., 2011).

### Pharmacokinetic Parameters of Probe Substrates

#### Midazolam

**FIGURE 2:**
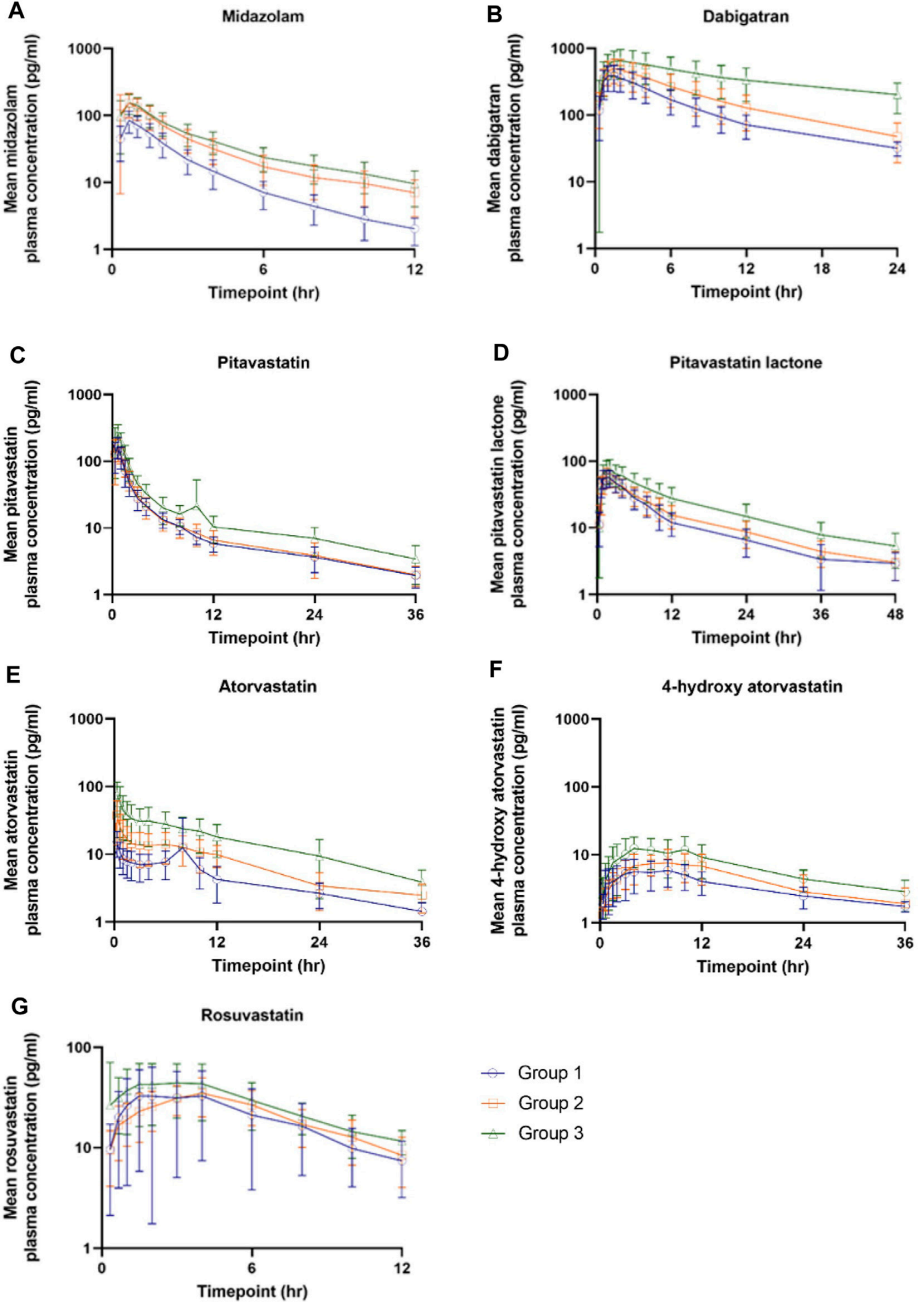
The plasma concentration-time curves of microdose cocktail probe substrates in three groups of participants **(A)** Midazolam **(B)** Dabigatran **(C)** Pitavastatin **(D)** Pitavastatin lactone **(E)** Atorvastatin **(F)** 4-hydroxy Atorvastatin, and **(G)** Rosuvastatin. Group 1: healthy young participants; Group 2: healthy elderly participants; Group 3: elderly patients with chronic kidney disease.

AUC_0-last_, AUC_0-inf_, and C_max_ of MDZ were significantly increased in healthy elderly (2.30, 2.32, and 1.88 fold, respectively) and elderly patients with CKD (2.90, 2.90, and 1.95 fold, respectively), as compared to healthy young adults. T_1/2_ was also prolonged in healthy elderly and elderly patients with CKD. No significant differences were seen between the groups of healthy elderly and elderly patients with CKD (Table 2; Figure 2A). As anticipated and previously shown by Prueksaritanont et al. (2017), RIF showed no effect on the pharmacokinetics of MDZ (Table 2).

**TABLE 2:** Pharmacokinetic parameters of midazolam and dabigatran.

Drugs	Healthy young adults			Healthy elderly		Elderly patients with chronic kidney disease	
	Microdose	Microdose + Rifampicin		Microdose		Microdose	
	GM (95%CI)	GM (95%CI)	GMR (95%CI)	GM (95%CI)	GMR (95%CI)	GM (95%CI)	GMR (95%CI)
**Midazolam**							
AUC_0-last_ (pg/mL.hr)	192 (155–237)	248 (198–309)	1.29 (0.96–1.74)	440 (342–567)	2.30 (1.70–3.09)*	556 (454–681)	2.90 (2.16–3.88)*
AUC_0-inf_ (pg/mL.hr)	200 (162–247)	256 (206–318)	1.28 (0.96–1.72)	464 (365–590)	2.32 (1.74–3.10)*	579 (475–705)	2.90 (2.18–3.85)*
C_max_ (pg/mL)	80 (67–96)	78 (96–118)	1.20 (0.92–1.56)	151 (119–191)	1.88 (1.44–2.44)*	156 (132–185)	1.95 (1.51–2.52)*
T_max_ (hr)^a^	0.7 (0.7–0.7)	0.7 (0.7–0.8)	−	0.7 (0.7–1.0)	−	0.7 (0.7–1.0)	−
T_1/2_ (hr)^a^	2.7 (2.1–3.4)	2.5 (1.8–3.0)	−	5.9 (4.0–7.7)*	−	7.3 (5.3–8.1)*	−
CL/F (mL/min/kg^0.75^)	127 (105–152)	98 (81–118)	0.77 (0.60–1.00)	53 (42–67)	0.42 (0.32–0.55)*	39 (32–47)	0.31 (0.23–0.40)*^,¶^
**Dabigatra**n							
AUC_0-last_ (pg/mL.hr)	2,661 (2,147–3,298)	6,228 (5,165–7,511)	2.34 (1.78–3.08)*	4,118 (3,136–5,408)	1.55 (1.13–2.12)*	10,930 (8,714–13,708)	4.11 (3.01–5.61)*^,¶^
AUC_0-inf_ (pg/mL.hr)	3,111 (2,577–3,757)	6,902 (5,849–8,146)	2.22 (1.74–2.83)*	4,557 (3,554–5,844)	1.46 (1.09–1.97)*	13,242 (10,480–16,732)	4.26 (3.18–5.70)*^,¶^
C_max_ (pg/mL)	375 (302–467)	699 (593–824)	1.86 (1.43–2.43)*	477 (378–601)	1.27 (0.94–1.73)	640 (506–808)	1.70 (1.26–2.30)*
T_max_ (hr)^a^	1.5 (1.0–1.5)	2.0 (2.0–2.5)*	−	1.5 (1.0–1.5)	−	1.5 (1.0–2.0)	−
T_1/2_ (hr)^a^	6.6 (4.6–7.8)	7.4 (5.8–10.6)	−	6.9 (5.9–8.2)	−	16.8 (14.5–21.7)*^,¶^	−
CL/F (mL/min/kg^0.75^)	228 (190–274)	97 (85–112)	0.43 (0.34–0.53)*	141 (109–184)	0.62 (0.47–0.83)*	49 (40–61)	0.22 (0.16–0.29)*^,¶^
CL_R_, mL/min	78 (70–88)	59 (53–66)	0.76 (0.65–0.88)*	48 (39–59)	0.61 (0.48–0.78)*	15 (12–18)	0.19 (0.15–0.24)*^,¶^

a Data are presented in the median (interquartile range).

*p-value <0.05, healthy young adult as a reference group.

^¶^*p*-value <0.05, healthy elderly as a reference group.

AUC_0-inf_: area under the concentration-time curve of time zero to infinity; AUC_0-last_: area under the concentration-time curve of time zero to the last time point; CI: confidence interval; CL/F: oral clearance; CL_R_: renal clearance; C_max_: maximum plasma concentration; GM: geometric mean; GMR: geometric mean ratio; T_max_: time to maximum plasma concentration; T_1/2_: half-life.

#### Dabigatran

AUC_0-last_ and AUC_0-inf_, but not C_max_ of DABI slightly increased with ageing (1.55, 1.46, and 1.27 fold, respectively), while elderly patients with CKD showed a marked increase in drug exposure (AUC_0-last_ 4.11 fold, AUC_0-inf_ 4.26 fold, and C_max_ 1.70 fold) when compared to healthy young controls. Elderly patients with CKD had higher AUC_0-last_ and AUC_0-inf_ than healthy elderly (GMR (95% CI): 2.65 (1.91–3.69) and 2.91 (2.14–3.95), respectively), but no differences were observed in C_max_. The theoretical AUCR of DABI was increased with the same magnitude, as seen in the observed AUCRs in healthy elderly (1.70 fold) but not for elderly patients with CKD (2.84 fold). Of note, both healthy elderly and elderly patients with CKD had a reduction in DABI CL_R_ by 40 and 80%, respectively, but only the elderly patients with CKD had a very prolonged DABI T_1/2_ (∼3 fold, Table 2 and Figure 2B). RIF significantly increased AUC_0-last_, AUC_0-inf_, and C_max_ of DABI but reduced CL_R_ of DABI by 25% (Table 2).

#### Pitavastatin and Pitavastatin Lactone

Only elderly patients with CKD showed a significant increase in AUC_0-last_, AUC_0-inf_, and C_max_ of PTV (1.67, 1.66, and 1.53 fold, respectively) and PTV-lactone (1.81, 1.87, and 1.35 fold, respectively), as compared to healthy young adults (Table 3; Figures 2C,D). On the other hand, the AUC_0-last_ ratios for PTV-lactone/PTV were similar in all groups of participants (Table 3). RIF markedly increased AUC_0-last_, AUC_0-inf_, and C_max_ of PTV, but lesser effects were observed with PTV-lactone, resulting in a significant decrease in the AUC_0-last_ ratios for PTV-lactone/PTV ratio when RIF was administered (Table 3).

**TABLE 3:** Pharmacokinetic parameters of pitavastatin and pitavastatin lactone.

Drugs	Healthy young adults			Healthy elderly		Elderly patients with chronic kidney disease	
	Microdose	Microdose + Rifampicin		Microdose		Microdose	
	GM (95%CI)	GM (95%CI)	GMR (95%CI)	GM (95%CI)	GMR (95%CI)	GM (95%CI)	GMR (95% CI)
**Pitavastatin**								
AUC_0-last_ (pg/mL.hr)	429 (375–491)	1,956 (1,656–2,312)	4.56 (3.71–5.61)*	446 (375–530)	1.04 (0.82–1.31)	715 (578–885)	1.67 (1.33–2.09)*,^¶^
AUC_0-inf_ (pg/mL.hr)	468 (405–540)	1,980 (1,677–2,339)	4.24 (3.42–5.24)*	494 (417–585)	1.06 (0.83–1.34)	774 (624–959)	1.66 (1.32–2.08)*^,¶^
C_max_ (pg/mL)	159 (132–191)	857 (729–1,008)	5.40 (4.25–6.86)*	154 (123–192)	0.97 (0.73–1.28)	242 (193–303)	1.53 (1.16–2.00)*^,¶^
T_max_ (hr)^a^	0.7 (0.7–0.7)	0.7 (0.7–0.7)	–	0.7 (0.7–0.7)	–	0.7 (0.7–0.7)	–
T_1/2_ (hr)^a^	12.2 (10.7–14.1)	6.5 (4.8–9.6)*	–	12.6 (11.4–15.9)	–	15.0 (11.3–17.6)	–
CL/F (mL/min/kg^0.75^)	19 (17–21)	4 (4–5)	0.22 (0.19–0.26)*	17 (15–20)	0.92 (0.74–1.13)	10 (8–12)	0.53 (0.43–0.65)*^,¶^
**Pitavastatin lactone**								
AUC_0-last_ (pg/mL.hr)	524 (440–623)	649 (556–759)	1.24 (0.99–1.56)	604 (494–739)	1.15 (0.89–1.50)	950 (767–1,177)	1.81 (1.41–2.34)*^,¶^
AUC_0-inf_ (pg/mL.hr)	574 (475–693)	690 (589–809)	1.20 (0.95–1.53)	676 (558–819)	1.18 (0.90–1.54)	1,071 (861–1,332)	1.87 (1.44–2.42)*^,¶^
C_max_ (pg/mL)	59 (51–68)	76 (67–86)	1.29 (1.08–1.55)*	48 (58–69)	0.98 (0.78–1.22)	80 (66–97)	1.35 (1.09–1.68)*^,¶^
T_max_ (hr)^a^	1.5 (1.0–1.8)	1.5 (1.0–2.0)	–	1.5 (1.5–1.5)	–	1.5 (1.5–2.0)	–
T_1/2_ (hr)^a^	13.1 (8.6–17.6)	12.8 (8.9–18.1)	–	13.7 (11.6–17.9)	–	15.3 (12.2–19.0)	–
**Pitavastatin lactone/** **P** **itavastatin ratio**								
AUC_0-last_	1.22 (1.08–1.38)	0.33 (0.30–0.37)	0.40 (0.34–0.47)*	1.35 (1.22–1.51)	1.13 (0.89–1.43)	1.33 (1.15–1.54)	1.13 (0.90–1.41)

^a^Data are presented in the median (interquartile range).

*p-value <0.05, healthy young adult as a reference group.

^¶^*p*-value <0.05, healthy elderly as a reference group.

AUC_0-inf_: area under the concentration-time curve of time zero to infinity; AUC_0-last_: area under the concentration-time curve of time zero to the last time point; CL/F: oral clearance; CI: confidence interval; C_max_: maximum plasma concentration; GM: geometric mean; GMR: geometric mean ratio; T_max_: time to maximum plasma concentration; T_1/2_: half-life.

#### Atorvastatin and 4-Hydroxy Atorvastatin

AUC_0-last_ and C_max_ of both ATV and 4-OH-ATV were increased in healthy elderly (2.14 and 2.22 fold, respectively, for ATV; 1.55 and 1.27 fold, respectively, for 4-OH-ATV) and more prominently increased in elderly patients with CKD (4.15 and 4.18 fold, respectively, for ATV; 2.58 and 1.84 fold, respectively, for 4-OH-ATV) as compared to healthy young participants (Table 4; Figures 2E,F). The AUC_0-last_ ratios for 4-OH-ATV/ATV were also reduced by approximately 20–30% in healthy elderly and elderly patients with CKD, compared to healthy adults (Table 4). RIF resulted in a marked increase in AUC_0-last_ and C_max_ of ATV and 4-OH-ATV, but no changes were observed in the AUC_0-last_ ratio for 4-OH-ATV/ATV (Table 4). Unfortunately, pharmacokinetic parameters of 2-OH-ATV could not be estimated as the plasma 2-OH-ATV concentrations were lower than the assay’s LLOQ.

**TABLE 4:** Pharmacokinetic parameters of atorvastatin, 4-hydroxy atorvastatin, rosuvastatin, and rosuvastatin with *ABCG2* gene wild-type (rs2231142).

Drugs	Healthy young adults			Healthy elderly		Elderly patients with chronic kidney disease	
	Microdose	Microdose + Rifampicin		Microdose		Microdose	
	GM (95%CI)	GM (95%CI)	GMR (95%CI)	GM (95%CI)	GMR (95%CI)	GM (95%CI)	GMR (95%CI)
**Atorvastatin**							
AUC_0-last_ (pg/mL.hr)	131 (102–168)	767 (609–965)	5.84 (4.21–8.10)*	281 (228–347)	2.14 (1.52–3.02)*	545 (411–725)	4.15 (2.98–5.79)*^,¶^
C_max_ (pg/mL)	16 (12–21)	258 (196–339)	16.37 (11.25–23.81)*	35 (27–45)	2.22 (1.49–3.31)*	66 (47–92)	4.18 (2.85–6.14)*^,¶^
T_max_ (hr)^a^	0.3 (0.3–0.3)	0.7 (0.7–1.0)*	–	0.3 (0.3–0.3)	–	0.3 (0.3–0.3)	–
CL/F (mL/min/kg^0.75^)	617 (479–793)	106 (88–126)	0.17 (0.13–0.23)*	274 (219–342)	0.44 (0.31–0.63)*	131 (100–173)	0.21 (0.15–0.30)*^,¶^
**4-Hydroxy atorvastatin**							
AUC_0-last_ (pg/mL.hr)	94 (74–120)	729 (590–901)	7.75 (5.67–10.58)*	146 (113–187)	1.55 (1.12–2.14)*	243 (194–304)	2.58 (1.88–3.53)*^,¶^
C_max_ (pg/mL)	7 (6–8)	109 (89–134)	16.08 (12.40–20.86)*	9 (7–11)	1.27 (0.94–1.72)	12 (9–17)	1.84 (1.37–2.47)*^,¶^
T_max_ (hr)^a^	5.0 (4.0–8.0)	2.0 (1.0–2.5)*	–	8.0 (6.0–10.0)	–	6.0 (4.0–10.0)	–
**4-Hydroxy atorvastatin/atorvastatin ratio**							
AUC_0-last_	0.72 (0.57–0.90)	0.95 (0.84–1.07)	1.20 (0.96–1.50)	0.52 (0.42–0.64)	0.78 (0.64–0.96)*	0.45 (0.37–0.53)	0.72 (0.59–0.87)*
**Rosuvastatin**							
AUC_0-last_ (pg/mL.hr)	195 (133–288)	851 (690–1,050)	4.35 (2.84–6.67)*	290 (228–369)	1.48 (0.95–2.32)*	372 (279–495)	1.90 (1.23–2.93)*
C_max_ (pg/mL)	32 (23–45)	232 (177–305)	7.21 (4.78–10.89)*	37 (31–45)	1.15 (0.78–1.69)	53 (40–70)	1.63 (1.12–2.37)*
T_max_ (hr)^a^	3.5 (1.8–4.0)	1.0 (1.0–1.5)*	–	4.0 (3.0–4.0)	–	2.0 (1.0–3.0)	–
CL/F (mL/min/kg^0.75^)	207 (144–297)	48 (39–57)	0.23 (0.15–0.34)*	133 (106–167)	0.64 (0.42–0.98)*	96 (73–127)	0.47 (0.31–0.70)*
CL_R_ (mL/min)	223 (192–259)	147 (131–164)	0.66 (0.55–0.79)*	157 (126–195)	0.70 (0.51–0.97)*	42 (30–60)	0.19 (0.14–0.26)*^,¶^
**Rosuvastatin with *ABCG2* gene wild-type (rs2231142)**							
AUC_0-last_ (pg/mL.hr)	136 (91–203)	700 (570–860)	5.15 (3.36–7.89)*	274 (180–419)	2.02 (1.16–3.51)*	285 (193–419)	2.09 (1.26–3.49)*
C_max_ (pg/mL)	25 (17–37)	203 (143–290)	8.04 (4.87–13.28)*	39 (33–47)	1.56 (0.97–2.50)	40 (30–52)	1.57 (1.01–2.44)*

^a^Data are presented in the median (interquartile range).

*p-value <0.05, healthy young adult as a reference group.

^¶^*p*-value <0.05, healthy elderly as a reference group.

AUC_0-last_: area under the concentration-time curve of time zero to the last time point; CI: confidence interval; CL/F: oral clearance; CL_R_: renal clearance; C_max_: maximum plasma concentration; GM: geometric mean; GMR: geometric mean ratio; T_max_: time to maximum plasma concentration; T_1/2_: half-life.

#### Rosuvastatin

Elderly patients with CKD showed increased AUC_0-last_ (1.90 fold) and C_max_ (1.63 fold) of RSV when compared to healthy young controls (Table 4; Figure 2G). The theoretical AUCRs of RSV were 1.19 fold in healthy elderly and 1.32 fold in elderly patients with CKD, compared to healthy young participants. CL_R_ of RSV was considerably low in elderly patients with CKD (80% reduction) compared to healthy elderly (30% reduction, Table 4). As previously observed (Prueksaritanont et al., 2017), AUC_0-last_ and C_max_ of RSV were increased with RIF (Table 4).

### Multivariate Analysis

Multivariate analyses for each drug in the microdose cocktail were carried out, adjusting for factors known to influence pharmacokinetic parameters. The results confirmed that all significant differences seen in the univariate analysis were independent of confounders (Table 5).

**TABLE 5:** Multivariate analysis of the differences in AUC_0-last_, C_max_, and T_1/2_ between three groups of participants.

Multivariate model	Midazolam	Dabigatran	Pitavastatin^a^	Pitavastatin lactone^a^	Atorvastatin^a^	4-Hydroxy atorvastatin^a^	Rosuvastatin^b^
N	53	53	52	52	52	52	29
**AUC_0-last_**							
Healthy young adults	1.00	1.00	1.00	1.00	1.00	1.00	1.00
Healthy elderly	2.19 (1.61–2.98)**	1.48 (1.06–2.05)*	0.99 (0.79–1.25)	1.25 (0.99–1.57)	2.00 (1.41–2.85)**	1.54 (1.10–2.14)*	2.02 (1.16–3.51)*
Elderly with CKD	2.68 (1.95–3.68)**	3.80 (2.72–5.31)**	1.57 (1.26–1.97)**	2.16 (1.70–2.74)**	4.15 (2.62–5.32)**	2.55 (1.82–3.56)**	2.09 (1.26–3.49)*
Elderly with CKD *vs* Healthy elderly	1.22 (0.89–1.67)	2.58 (1.85–3.59)**	1.59 (1.26–2.01)**	1.73 (1.37–2.18)**	1.86 (1.30–2.66)*	1.66 (1.17–2.34)*	1.04 (0.57–1.88)
Body weight	–	–	–	0.98 (0.98–0.99)**	–	–	–
Albumin	–	–	0.64 (0.41–1.00)*	–	–	–	–
Total bilirubin	–	–	–	0.79 (0.64–0.99)*	–	–	–
**C_max_**							
Healthy young adults	1.00	1.00	1.00	1.00	1.00	1.00	1.00
Healthy elderly	1.83 (1.40–2.38)**	1.30 (0.99–1.71)	1.02 (0.80–1.31)	1.06 (0.89–1.27)	2.15 (1.41–3.27)*	1.32 (0.97–1.79)	1.69 (1.11–2.55)*
Elderly with CKD	1.86 (1.42–2.44)**	1.91 (1.43–2.56)**	1.78 (1.38–2.30)**	1.63 (1.35–1.96)**	3.98 (2.61–6.08)**	1.71 (1.26–2.33)*	1.92 (1.28–2.87)*
Elderly with CKD *vs* Healthy elderly	1.02 (0.77–1.34)	1.47 (1.10–1.96)*	1.74 (1.36–2.24)**	1.53 (1.28–1.84)**	1.85 (1.21–2.83)*	1.30 (0.94–1.79)	1.14 (0.73–1.78)
Body weight	–	0.98 (0.97–0.99)**	0.98 (0.97–0.99)**	0.98 (0.98–0.99)**	–	–	0.98 (0.97–0.99)*
**T_1/2_**							
Healthy young adults	1.00	1.00					
Healthy elderly	2.31 (1.72–3.10)**	1.15 (0.93–1.44)					
Elderly with CKD	2.52 (1.89–3.36)**	2.93 (2.35–3.64)**					
Elderly with CKD *vs* Healthy elderly	1.09 (0.80–1.48)	2.54 (2.01–3.19)**					

Data are presented in the geometric mean ratio (95% confidence interval).

Variables with a *p*-value < 0.1 in the univariate analysis were included in the multivariable analysis.

a A subject with SLCO1B1 wild-type’s plasma drug concentration was an outlier and was excluded from the model.

b Twenty four subjects with ABCG2 variants (rs2231142, 421AA and 421CA) were excluded from the model.

*p < 0.05, **p < 0.001.

AUC_0-last_: area under the concentration-time curve of time zero to the last time point; C_max_: maximum plasma concentration; CKD: chronic kidney disease; T_1/2_: half-life.

### Effects of Genetic Variations on the Pharmacokinetics of Statins and Midazolam

The genetic variations of the *SLCO1B1* gene did not affect the pharmacokinetics of statins used in this study. In contrast, participants with *ABCG2* variants (421AA and 421CA genotypes, *n* = 24) showed increased AUC_0-last_ and C_max_ of RSV than *ABCG2* wild type (Supplementary Table S5). Therefore, the participants with genotype variants (421AA and 421CA) were excluded from the multivariate model in a sensitivity analysis. The results showed that AUC_0-last_ and C_max_ of RSV in the elderly patients with CKD and AUC_0-last_ in healthy elderly were higher than those corresponding values in the healthy young participants (Table 4 and Supplementary Figure S1).

Due to the non-significant correlation of *CYP3A* genetic variation with MDZ pharmacokinetics as reported in previous studies (He et al., 2005; Miao et al., 2009; Singh et al., 2009), CYP3A5*3 genetic variation was not included in multivariate analysis. Supplementary Table S3 showed the genotype frequency of CYP3A5*3. Pharmacokinetics of MDZ in wild-type *CYP3A5* showed an increase in AUC_0-last_ (2.53 fold), AUC_0-inf_ (2.57 fold), and C_max_ (1.83 fold) in elderly patients with CKD as compared to healthy young participants (Supplementary Table S6). Pharmacokinetics profiles of MDZ between *CPY3A5* genetic variants (6968AG and 6986GG) and wild type (6986AA) were also compared in healthy young participants. There were no differences observed between them (Supplementary Table S7).

### Safety

Mild adverse events including diarrhea, dysmenorrhea, dyspepsia, migraine, and nasal congestion were observed in six participants. These adverse events were unrelated to the drugs listed in the microdose cocktail. In study period 2, healthy young participants had darkened urine as a result of RIF administration. Additionally, there were no significant changes in the physical examination and clinical laboratory parameters between baseline and the end of study (Supplementary Table S8).

## Discussion

Our study investigated the activity of CYP3A and drug transporters in three participant groups using a validated microdose cocktail containing five probe substrates, with no inhibitor activity of any enzymes and/or transporters (U.S. Food and Drug Administration, 2020). We found that CYP3A and intestinal P-gp activities are reduced in ageing and CKD, respectively. There is a trend of changes in OATP1B and BCRP activity measured by microdose cocktail probe drugs.

The probe substrates in the drug cocktail should be specific and devoid of any drug-drug interactions to allow precise quantification of enzymes or activity of drug transporters (Fuhr et al., 2019). Our microdose cocktail had specificity (MDZ) and relative selectivity (DABE and PTV). The microdose cocktail has been validated in Caucasians, showing a potential of simultaneous assessment for enzyme and drug transporter activity (Prueksaritanont et al., 2017). Here, we verified the utilization of the cocktail in the Thai population. Our microdose cocktail-RIF drug-drug interaction study in the Thai population showed similar findings as reported in the earlier study (Prueksaritanont et al., 2017).

Reductions in CYP3A activity were observed with similar magnitude in both elderly with or without CKD, shown by the increased AUC and C_max_ and prolonged T_1/2_ of MDZ. It should be noted that oral MDZ as a probe drug represents CYP3A activity in both intestine and liver and that the change in MDZ pharmacokinetics found in this study could be attributed to changes in intestinal and/or hepatic CYP3A activity. Additionally, as MDZ is an intermediate hepatic extraction drug, a physiological reduction of hepatic blood flow anticipated in the elderly might also contribute to the observed reduction in hepatic clearance of MDZ (Dundee et al., 1986; Amrein and Hetzel, 1990). Our finding that CKD was not associated with changes in pharmacokinetic profiles of MDZ was consistent with a previous study where neither AUC nor C_max_ of MDZ was affected by renal impairment (Tatosian et al., 2020).

Genetic polymorphisms of *the CYP3A* gene were reported in the Thai population (Mauleekoonphairoj et al., 2020). *CYP3A4* polymorphisms are rarely found in Asians, whereas 65–85% of Asians have CYP3A5*3 (Lamba et al., 2002; Hutchison and O'Brien, 2007; Zhou et al., 2017; Mauleekoonphairoj et al., 2020). Although some studies indicated that polymorphisms of CYP3A4/5 might affect the clearance of their substrate such as tacrolimus, the genetic variation in CYP3A enzymes has no significant correlation with *in vivo* MDZ metabolism and disposition (He et al., 2005; Miao et al., 2009; Singh et al., 2009). Here, CYP3A5*3 genotyping and pharmacokinetic parameters analysis were also assessed (Supplementary Table S3). The results showed that elderly patients with CKD who were with wild-type *CYP3A5* (*n* = 5) had increased AUC and C_max_ of MDZ (GMR∼2.0–3.0) when compared to healthy young participants with wild-type *CYP3A5* (*n* = 5, Supplementary Table S6). This magnitude of increases in AUC and C_max_ of MDZ in participants with wild-type *CYP3A5* was similar to that in all other participants studied (Table 2). The comparison between wild-type and variant genotypes of *CYP3A5* within the group of healthy young participants was also analyzed, and no significant difference was observed (Supplementary Table S7). Therefore, the genetic variations of the CYP3A5 enzyme did not affect the pharmacokinetics of MDZ in our study.

A widely used probe substrate for an intestinal efflux transporter P-gp is DABE. DABE is rapidly converted to DABI after absorption (Chu et al., 2018). The reduction of P-gp activity may decrease the efflux of DABE in the intestine, increasing plasma DABI levels (Chu et al., 2018). In this study, the AUC of DABI was increased in the elderly both with and without CKD compared to healthy young adults. Considering that DABI is 80% excreted into the urine in an unchanged form (Muñoz-Corcuera et al., 2016), CL_R_ of DABI was measured. The magnitude of DABI’s AUC augmentation was prominent in elderly patients with CKD, corresponding with a more significant CL_R_ reduction (Table 2). Compared to healthy young participants, the theoretical AUCRs for DABI in healthy elderly and elderly patients with CKD were increased by 1.47 and 2.84 fold, respectively. These data suggested that ageing may not affect intestinal P-gp function since the observed AUCR for DABI of healthy elderly in our study (1.46 fold, Table 2) was closed to the theoretical AUCR (1.47 fold). However, intestinal P-gp is possibly affected by CKD as the observed AUCR for DABI in elderly patients with CKD (4.26 fold, Table 2) is much higher than the theoretical AUCR (2.84 fold). By this, intestinal P-gp function might be impaired, leading to an approximately 1.42 fold increase in AUCR for DABI in elderly patients with CKD. Likewise, a previous study also reported no significant AUC elevation of digoxin, another probe substrate of P-gp at intestinal and systemic levels in the elderly, compared to young adults (Larsen et al., 2007). Reports in a rat model also suggested a downregulation of intestinal P-gp activity in CKD associated with increased drug absorption (Veau et al., 2001; Naud et al., 2007). The latest clinical study in CKD (Tatosian et al., 2020) observed a progressive increase in AUC_0-inf_ and C_max_ of DABI with increasing severity of renal impairment up to 4.9 and 1.7 fold, respectively, which is in keeping with our data. Their findings were confirmed with the co-administration of RIF. Here, we also showed that elderly patients with CKD had increased AUC of DABI and the change in AUCRs supports a reduction of intestinal P-gp activity in renal failure. However, unlike the AUC, an increase in C_max_ of DABI in elderly patients with CKD compared to healthy elderly did not reach statistical significance. Hence, the intestinal P-gp activity in CKD might not be meaningfully affected.

OATP1B is an uptake drug transporter in the liver, where PTV is a probe substrate for this transporter (Prueksaritanont et al., 2014). OATP1B transfers PTV from the portal vein into hepatocytes. PTV is not a significant substrate of human CYP450 but mainly undergoes rapid glucuronidation by UDP-glycosyltransferase 1A1 (UGT1A1), UGT1A2, and UGT2B7 to PTV-lactone. Unlike PTV, PTV lactone is not a substrate for OATP1B (Alagona, 2010). The reduction of OATP1B activity can increase plasma drug levels of an OATP1B substrate (Shitara, 2011). In our study, only OATP1B activity in the elderly patients with CKD declined compared to healthy young participants and healthy elderly, as demonstrated by increased AUC and C_max_ of PTV (Table 3). This finding is consistent with a previous pharmacokinetic study in patients with non-hemodialysis moderate and severe renal impairment (Morgan et al., 2012). A reduction in OATP1B transporter expression and activity was reported in CKD rat models (Sun et al., 2006; Naud et al., 2008). Additionally, a physiologically-based pharmacokinetic (PBPK) study showed a 60% reduction in hepatic OATP1B transporter activity in patients with severe CKD (Tan et al., 2019). Our findings were different from Tatosian, et al. (Tatosian et al., 2020) as we also observed an increase in AUC and C_max_ of PTV-lactone, and no differences were found with the AUC_0-last_ of PTV-lactone/PTV ratio in the elderly patients with CKD (Table 3). The underlying reasons for the discrepancy between the two studies remain unclear.

BCRP is an intestinal efflux transporter capable of reducing systemic exposure of many oral drugs and substrates, including RSV (Huang et al., 2006). Consistent with other studies (Zhang et al., 2006; Keskitalo et al., 2009; Lee et al., 2013), we found that polymorphisms of *ABCG2* affected pharmacokinetic profiles of RSV as evidenced by increased AUC and C_max_ of RSV in participants with *ABCG2* variant (Supplementary Table S5). After excluding the participants with this genetic confounder, we observed a significant increase in AUC of RSV in healthy elderly and a more prominent increase of AUC in elderly patients with CKD (Table 4). In addition, the observed AUCRs of RSV in healthy elderly and elderly patients with CKD when compared to healthy young adults were 2.02 and 2.09 fold, respectively (Table 4), which were higher than the theoretical AUCRs of RSV (1.19 fold in healthy elderly and 1.32 fold in elderly patients with CKD). This finding suggested that BCRP transporter activity might be affected by both ageing and CKD. However, a more significant effect on C_max_ of RSV in healthy elderly and elderly patients with CKD should have occurred if intestinal BCRP transporter activity was reduced (Table 4). Hence, an association of ageing and CKD with BCRP represented by changes in pharmacokinetic profiles of RSV still cannot be entirely determined in our study. Other studies reported no association between ageing and RSV exposure (Martin et al., 2002; Davidson, 2007; Kim et al., 2019). Our data is consistent with Tatosian, et al. (Tatosian et al., 2021) where RSV pharmacokinetics alteration was not associated with CKD. In addition, RSV is another substrate of hepatic OATP1B transporter but is less sensitive and less selective than PTV (Prueksaritanont et al., 2014). Therefore, when considering the pharmacokinetic changes of RSV with PTV results as discussed above, a definitive effect of CKD on hepatic OATP1B transporter activity is still yet to be concluded.

ATV is mainly metabolized by CYP3A and transported by hepatic OATP1B (major), intestinal BCRP, and possibly, to a minor extent, intestinal P-gp (Kellick et al., 2014; Kellick, 2017). Here, we found a significant increase in AUC and C_max._ of ATV associated with ageing and CKD. The pharmacokinetic changes of ATV were in support of the pharmacokinetic changes seen with MDZ. CYP3A4 converts ATV into its major metabolite, 4-OH-ATV (Hoffart et al., 2012). A reduction in the AUC_0-last_ ratio for 4-OH-ATV/ATV and the association of ageing and changes in ATV plasma levels observed in this study (Table 4) was in line with findings observed with MDZ on CYP3A. The effect of ageing on pharmacokinetic profiles of ATV (Table 4) is similar to a previous report of reduced CYP3A enzyme activity in ageing (Gibson et al., 1996).

On the other hand, the effects of CKD on pharmacokinetic profiles of ATV and its metabolite in our study were inconclusive. In the recent study by Tatosian, et, al., increases in AUC and C_max_ of ATV were associated with the severity of renal impairment (Tatosian et al., 2020). Therefore, P-gp and BCRP may be additional factors involved in alterations of ATV pharmacokinetics in elderly patients with CKD. Unfortunately, BCRP activity was inconclusive in our study due to the results of RSV as discussed above.

It is important to note that although age- and CKD-associated changes in OATP1B and BCRP drug transporter activity could not be definitively concluded, our results suggest a trend in their changes. Furthermore, these findings are potentially beneficial to clinicians as most older people are generally taking multiple drug treatments and are therefore at risk of drug-drug interactions.

It is also worth noting that our study did not directly measure unbound plasma concentrations due to the assay sensitivity limitation. However, plasma protein binding was indirectly determined by *ex-vivo* experiments using pooled predose plasma samples from each study group, spiked with relevant plasma concentrations of substrates (5–30 ng/ml). No significant difference of unbound fraction amongst the three studied groups was found for all drugs used in this cocktail (data not shown). These data suggested a negligible effect of plasma protein binding in our study, and consequently, similar conclusions would be expected with unbound drug pharmacokinetics. Currently, efforts are underway to develop a PBPK model using data from this study to delineate the effect of ageing and CKD on the underlying mechanisms related to drug metabolizing enzymes and drug transporters in elderly patients with CKD.

This study has limitations. In general, gender could affect the pharmacokinetics of drugs because of the differences in their physiology. For instance, women usually have lower body weight, lower muscle mass, and less intestinal enzymatic activity than men (Whitley and Lindsey, 2009). Although participants in this study were not intentionally matched by gender, which is one of our limitations, the significant difference in pharmacokinetic parameters was not found after gender adjustment in the multivariable analysis. Total body weight determines body composition (lean weight: adipose weight), affecting the volume distribution of drugs (Muñoz-Corcuera et al., 2016). A significant difference in body weight was seen at baseline between healthy young participants and elderly patients with CKD, but no effect of body weight on pharmacokinetic of drugs was found in the multivariate analysis. We have also carried out a subgroup analysis stratified by severity of CKD, and there was no significant difference shown between moderate and severe renal impairment (data not shown). No potential pharmacokinetic drug-drug interaction was observed between concomitant drugs and probe drugs. However, the screening for pharmacodynamics drug-drug interaction has not been performed.

Additionally, further studies with larger cohort sizes that stratify based on CKD severity, together with patients on dialysis and RIF co-administration in all study populations, may be warranted. Population pharmacokinetic studies would be helpful to elucidate if and how other factors affect the pharmacokinetics of the probe drugs profiles. Finally, PBPK studies using data from this study would provide more information to inform predictions of changes in drug metabolizing enzymes and drug transporter activity in elderly patients with CKD.

In conclusion, the microdose cocktail approach provides a valuable and safe screening tool to determine pharmacokinetic parameter alterations showing reduced CYP3A activity in the elderly. Intestinal P-gp activity might be reduced in CKD, but as the results in AUCR and C_max_ were conflicting, this has to be confirmed in further studies. Health care providers should be aware of the potential consequences of administering CYP3A and intestinal P-gp substrates or inhibitors to these target populations.

## Data Availability

The original contributions presented in the study are included in the article/Supplementary Material, further inquiries can be directed to the corresponding author.
