# Supplementary material for: Quantification of CYP3A and Drug Transporters Activity in Healthy Young, Healthy Elderly and Chronic Kidney Disease Elderly Patients by a Microdose Cocktail Approach
Source: Front Pharmacol. 2021 Sep 17;12:726669. doi: 10.3389/fphar.2021.726669 (PMC8486002; doi:10.3389/fphar.2021.726669)
Supplement: Supplementary file 1 [file DataSheet1.pdf]

Supplementary Material

Supplementary Table 1. Inclusion and exclusion criteria of the study.

	**Healthy young adults**	**Healthy elderly**	**Elderly with CKD**
**Inclusion criteria**			
	Thai male or female, aged 20 to 40 years (inclusive)	Thai male or female aged $\geq$60 years	
	Body mass index 18-25 kg/m^2 (inclusive)	Body mass index 18-30 kg/m^2 (inclusive)	
	Healthy by medical history, physical examination, and vital signs		Diagnosed with CKD according to the Kidney Disease Improving Global Outcomes 2012 criteria
	Laboratory values at screening visit[a] are within the normal range or showing no clinically significant abnormalities as confirmed by the clinical investigator		
	eGFR[b] $\geq$90 mL/min/1.73 m^2	eGFR[b] >60 mL/min/1.73 m^2	eGFR[b] 15-60 mL/min/1.73 m^2
**Exclusion criteria**			
	History of drug allergy to midazolam, dabigatran etexilate, pitavastatin, atorvastatin, rosuvastatin, and rifampicin		
	History of any illness that, in the opinion of the clinical investigator, might confound the result of the study or pose an additional risk in administrating the study drug to the subjects. This may include but is not limited to: a history of relevant drug or food allergies; history or cardiovascular disease, gastrointestinal, central nervous system disease, renal and hepatic impairment; history or presence of clinically significant illness; or history of mental illness that may affect compliance with study requirements	History of hypertension, diabetes mellitus, other major systemic diseases	History of renal transplantation, liver transplantation, hemodialysis and peritoneal dialysis
	Receive any medical prescription within 14 days before the first administration of the microdose cocktail, especially drug related to the study drug metabolizing enzyme		-
	History of heavy smoking (> 10 cigarettes per day) or moderate smoking (< 10 cigarettes per day) and cannot omit smoking a least one day before the study and until the completion of the study phase		
	History of alcoholic (more than 2 years) or moderate drinker (more than 3 drinks per day) or history of any drug abuse		
	Pregnant or breastfeeding (female)	-	-

[a]Laboratory tests include complete blood count, fasting blood sugar, blood urea nitrogen, serum creatinine, alkaline phosphatase, alanine aminotransferase, aspartate aminotransferase, total bilirubin, direct bilirubin, total protein, albumin, total cholesterol, triglyceride, low-density lipoprotein cholesterol, high-density lipoprotein cholesterol, electrolytes, parathyroid hormone, and phosphate.

[b]Modification of Diet in Renal Disease estimated glomerular filtration rate for Thai formula (Praditpornsilpa K, *et al*. The need for robust validation for MDRD-based glomerular filtration rate estimation in various CKD populations. Nephrol Dial Transplant. 2011 Sep;26(9):2780-5)

CKD, chronic kidney disease; eGFR, estimated glomerular filtration rate.

Supplementary Table 2. Geometric means (95% confidence interval) of pharmacokinetic parameters between midazolam powder formulation and midazolam solution formulation in elderly with chronic kidney disease.

PK parameters	Powder formulation (n = 13)	Solution formulation (n = 4)
$AUC_{0\text{-}last}$ (pg/mL.hr)	441.13 (323.81, 600.95)	437.37 (193.88, 986.69)
C_{max} (pg/mL)	150.06 (111.23-202.46)	152.84 (118.73, 196.75)
T_{max} (hr)[a]	0.67 (0.67-0.67)	1.00 (0.33-1.00)
$T_{1/2}$ (hr)[a]	5.50 (3.38-5.99)	6.46 (6.21-10.93)

[a]Data are presented in the median (interquartile range).

$AUC_{0\text{-}last}$: area under the concentration-time curve of time zero to the last time point; C_{max}: maximum plasma concentration; T_{max}: time to maximum plasma concentration; $T_{1/2}$: half-life.

Supplementary Table 3. Genotype frequency of *CYP3A5*3* (rs776746, 6986A > G) in three groups of participants

Genotype frequency (n)	Healthy young adults (n=20)	Healthy elderly (n=16)	Elderly patients with chronic kidney disease (n=17)	Total (n=53)
rs776746 (6986A > G)[a]				
6986AA (wild-type)	3	0	5	8
6986AG	9	8	5	22
6986GG	8	8	7	23

[a]Venous blood sample (3 mL) was collected from each participant into an EDTA tube. Genomic DNA was extracted by PureLink® Genomic DNA kit (ThermoFisher Scientific). *CYP3A5*3* c.6986A>G (rs776746) was detected by TaqMan® Real-time Polymerase Chain Reaction (PCR) using TaqMan Drug Metabolism Genotyping Assays (Applied Biosystem, ThermoFisher Scientific). PCR was performed with the quantitative real-time PCR (q-PCR) system (StepOnePlus™ Real-Time PCR System, ThermoFisher Scientific).

Supplementary Table 4. List of concomitant medication in elderly patients with chronic kidney disease

Concomitant drugs	Number of patients
Alfuzosin	2
Allopurinol	7
Amlodipine	5
Aspirin	5
Atenolol	2
Benzbromarone	1
Betahistine	1
Calciferol	6
Calcium carbonate	3
Candesartan	1
Carvedilol	2
Cetirizine	1
Clopidogrel	1
Colchicine	3
Diacerein	1
Doxazosin	3
Dutasteride	1
Enalapril	5
Eprex	1
Ezetimibe	1
Ferrous sulfate	4
Folic acid	7
Furosemide	4
Gabapentin	1
Glipizide	3
Hemax injection	2
Hydralazine	1
Insulin mixtard HM30	1
Insulin novo mix penfill injection	1
Irbesartan	2
Lercanidipine	1
Linagliptin	3
Lorazepam	1
Losartan	4
Manidipine	6
Melatonin	1
Metoprolol	1
Nicergoline	1
Omeprazole	2
Perindopril	1
Pioglitazone	2
Pocitrin (K, Na)	1
Pregabalin	1
Propranolol	1
Sennoside b	1
Silodosin	1
Simethicone	1
Simvastatin	6
Sodamint	7
Sodium chloride	1
Vitamin B6	1

Supplementary Table 5. Multivariable analysis of rosuvastatin with genetic variation.

Multivariate model	Rosuvastatin[a]
**n**	52
**$AUC_{0\text{-last}}$ (pg/mL.hr)**	
Healthy young adults	1.00
Healthy elderly	1.43 (0.95-2.16)[ns]
Elderly with CKD	1.83 (1.25-2.69)*
Elderly with CKD *vs* Healthy elderly	1.28 (0.85-1.93)[ns]
ABCG2 wild-type	1.00
ABCG2 421AA	3.06 (1.51-6.18)*
ABCG2 421CA	1.67 (1.18-2.35)*
**C_{max} (pg/mL)**	
Healthy young adults	1.00
Healthy elderly	1.12 (0.77-1.64)[ns]
Elderly with CKD	1.59 (1.12-2.26)*
Elderly with CKD *vs* Healthy elderly	1.42 (0.97-2.07)[ns]
SLCO1B1 wild-type	-
*SLCO1B1 *15/*17*	-
ABCG2 wild-type	1.00
ABCG2 421AA	2.18 (1.14-4.16)*
ABCG2 421CA	1.43 (1.04-1.96)*

Data are presented in the geometric mean ratio (95% confidence interval).
Variables with p-value <0.1 in the univariate analysis were included in the multivariable analysis.
[a]24 subjects with *ABCG2* variants (rs2231142, 421AA, and 421CA) were included in the model.
*p <0.05
$AUC_{0\text{-last}}$: area under the concentration-time curve of time zero to the last time point; C_{max}: maximum plasma concentration; ns: non-significant.

Supplementary Table 6. Pharmacokinetic parameters of midazolam in wild-type *CYP3A5* participants

Drugs	Healthy young adults (n=3)			Healthy elderly (n=0)		Elderly patients with chronic kidney disease (n=5)	
	Microdose	Microdose + Rifampicin		Microdose		Microdose	
	GM (95% CI)	GM (95% CI)	GMR (95% CI)	GM (95% CI)	GMR (95% CI)	GM (95% CI)	GMR (95% CI)
**Midazolam**							
$AUC_{0\text{-last}}$ (pg/mL.hr)	244 (140-424)	344 (183-646)	1.41 (0.82-2.42)	-	-	619 (411-931)	2.53 (1.49-4.31)*
$AUC_{0\text{-inf}}$ (pg/mL.hr)	254 (136-473)	360 (195-665)	1.42 (0.81-2.49)	-	-	653 (435-980)	2.57 (1.49-4.42)*
C_{max} (pg/mL)	95 (89-102)	119 (111-128)	1.26 (1.18-1.34)*	-	-	174 (144-210)	1.83 (1.46-2.28)*
T_{max} (hr)[a]	0.7 (0.7-1.0)	0.7 (0.7-1.5)	-	-	-	0.7 (0.7-1.0)	-
$T_{1/2}$ (hr)	2.9 (0.9-8.7)	4.7 (0.5-42.2)	1.65 (0.34-8.06)	-	-	8.4 (5.2-13.7)	2.96 (1.42-6.13)*

[a]Data are presented in the median (interquartile range).
*p-value <0.05, healthy young adult as a reference group.
¶p-value <0.05, healthy elderly as a reference group.
$AUC_{0\text{-inf}}$: area under the concentration-time curve of time zero to infinity; $AUC_{0\text{-last}}$: area under the concentration-time curve of time zero to the last time point; CI: confidence interval; C_{max}: maximum plasma concentration; GM: geometric mean; GMR: geometric mean ratio; T_{max}: time to maximum plasma concentration; $T_{1/2}$: half-life.

Supplementary Table 7. Pharmacokinetic parameters of midazolam in healthy young adults (n=20) categorized by *CYP3A5* genotypes.

Drugs	6986AA (wild-type, n=3)	6986AG (n=9)		6986GG (n=8)	
	GM (95% CI)	GM (95% CI)	GMR (95% CI)	GM (95% CI)	GMR (95% CI)
**Midazolam**					
$AUC_{0\text{-last}}$ (pg/mL.hr)	244 (140-424)	177 (111-280)	0.72 (0.38-1.40)	192 (147-252)	0.78 (0.40-1.53)
$AUC_{0\text{-inf}}$ (pg/mL.hr)	254 (136-473)	184 (117-289)	0.72 (0.38-1.38)	200 (153-261)	0.79 (0.41-1.52)
C_{max} (pg/mL)	95 (89-102)	77 (51-118)	0.82 (0.46-1.45)	78 (63-97)	0.82 (0.46-1.47)
T_{max} (hr)[a]	0.7 (0.7-1.0)	0.7 (0.7-0.7)	-	0.7 (0.7-0.7)	-
$T_{1/2}$ (hr)	2.9 (0.9-8.7)	2.3 (1.5-3.4)	0.79 (0.43-1.44)	2.9 (2.4-3.6)	1.02 (0.55-1.88)

[a]Data are presented in the median (interquartile range).

*p-value <0.05, 6986AA wild-type as a reference group.

$AUC_{0\text{-inf}}$: area under the concentration-time curve of time zero to infinity; $AUC_{0\text{-last}}$: area under the concentration-time curve of time zero to the last time point; CI: confidence interval; C_{max}: maximum plasma concentration; GM: geometric mean; GMR: geometric mean ratio; T_{max}: time to maximum plasma concentration; $T_{1/2}$: half-life.

Supplementary Table 8. Safety laboratory parameters on first and last visits.

Parameters	Healthy young adults		Healthy elderly		Elderly patients with chronic kidney disease	
	First visit	Last visit	First visit	Last visit	First visit	Last visit
Blood urea nitrogen (mg/dL)	10 (10-12)	11 (9-13)	11 (10-14)	12 (11-16)	26 (21-37)	28 (20-42)
Serum creatinine (g/dL)	0.7 (0.7-0.9)	0.7 (0.7-0.9)	0.7 (0.6-0.8)	0.6 (0.6-0.7)	2.2 (1.8-2.9)	2.0 (1.5-2.8)
eGFR corrected by BSA (mL/min)	112 (106-118)	116 (106-118)	95 (86-110)	110 (100-123)	33 (24-40)	38 (25-49)
Parathyroid hormone (pg/mL)	55 (45-60)	52 (46-59)	63 (49-76)	42 (35-66)	113 (76-168)	135 (101-166)
Fasting plasma glucose (mg/dL)	85 (81-93)	85 (80-88)	95 (90-102)	93 (89-102)	98 (88-105)	98 (83-118)
Total protein (g/dL)	7.8 (7.5-7.9)	7.5 (7.0-8.0)	7.7 (7.3-7.8)	7.4 (7.0-8.1)	7.6 (7.1-7.7)	7.6 (7.2-7.6)
Albumin (g/dL)	4.5 (4.3-4.6)	4.5 (4.3-4.8)	4.3 (4.3-4.5)	4.4 (4.0-4.5)	4.3 (4.2-4.4)	4.2 (4.0-4.3)
Total bilirubin (mg/dL)	0.6 (0.5-0.8)	0.4 (0.3-0.8)	0.6 (0.6-0.9)	0.6 (0.5-0.7)	0.6 (0.5-0.7)	0.6 (0.5-0.6)
Direct bilirubin (mg/dL)	0.2 (0.2-0.4)	0.2 (0.2-0.3)	0.3 (0.2-0.3)	0.2 (0.2-0.3)	0.3 (0.2-0.3)	0.2 (0.2-0.3)
Aspartate aminotransferase (U/L)	16 (15-20)	15 (14-17)	22 (19-24)	20 (16-21)	18 (16-26)	17 (14-28)
Alanine aminotransferase (U/L)	15 (13-20)	13 (10-18)	19 (16-22)	17 (14-20)	19 (16-25)	17 (13-27)
Alkaline phosphatase (U/L)	54 (47-66)	54 (46-64)	67 (56-75)	65 (60-77)	64 (53-73)	63 (53-73)
Total cholesterol (mg/dL)	203 (176-225)	196 (172-214)	242 (209-270)	228 (198-256)	167 (152-177)	174 (142-210)
Triglyceride (mg/dL)	87 (61-107)	94 (78-136)	130 (94-191)	113 (78-189)	91 (87-141)	103 (73-124)

Data are presented in the median (interquartile range). BSA: body surface area; eGFR: estimated glomerular filtration rate.

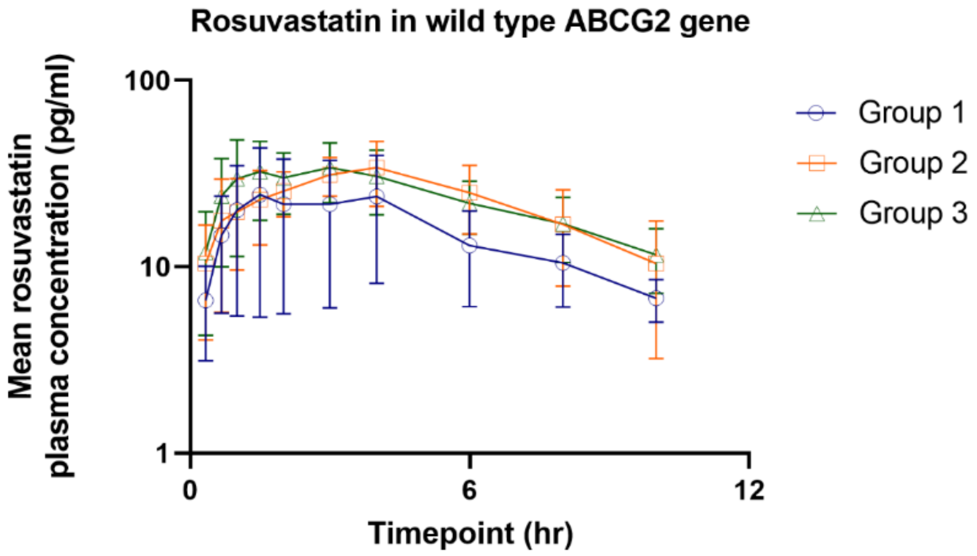

Supplementary Figure 1. Plasma concentration-time curve of rosuvastatin of wild-type *ABCG2* gene in 3 groups of participants.
Group 1: healthy young subjects
Group 2: healthy elderly subjects
Group 3: elderly patients with chronic kidney disease
